# Beyond refractive error: myopia’s exponential burden on retinal health with each diopter

**DOI:** 10.1186/s40942-025-00745-7

**Published:** 2025-11-04

**Authors:** Leo Arnal, Yeabsira Mesfin, Christine Xu, Anish Salvi, Kapil Mishra, Chase A. Ludwig

**Affiliations:** 1 Department of Ophthalmology, Byers Eye Institute, Horngren Family Vitreoretinal Center, Stanford University School of Medicine, 2452 Watson Court, Palo Alto, CA 94303 USA; 2 School of Medicine, University of California, San Francisco School of Medicine, 533 Parnassus Ave, San Francisco, CA 94143 USA; 3 Department of Ophthalmology, University of California, Irvine School of Medicine, 1001 Health Sciences Rd, Irvine, CA 92617 USA

**Keywords:** Myopia, Retina, Choroidal neovascularization, Myopic macular degeneration, Foveoschisis, Macular hole, Rhegmatogenous retinal detachment, Foveal retinal detachment

## Abstract

**Background:**

As myopia reaches epidemic levels worldwide, its role in driving vision-threatening retinal complications is increasingly urgent. This study quantifies the burden of myopia by examining its association with key retinal diseases and how risk escalates with increasing severity.

**Methods:**

We conducted a retrospective cohort study using the STARR clinical data warehouse, including all patients with ≥ 1 documented eye visit. Myopia severity was defined by spherical equivalent and axial length, classifying patients as non-myopic, myopic, highly myopic, or severely myopic. Primary outcomes included six retinal diseases associated with myopia: choroidal neovascularization (CNV), myopic macular degeneration (MMD), foveoschisis, macular hole (MH), rhegmatogenous retinal detachment (RRD), and foveal retinal detachment (FRD). Adjusted logistic regression estimated odds by myopia severity and spherical equivalent. Mean age at diagnosis was compared across groups.

**Results:**

Retinal complications occurred at younger ages with increasing myopia severity. Compared to non-myopes, myopic, highly myopic, and severely myopic patients had 2.45 (95% CI: 2.36–2.55), 2.46 (95% CI: 2.31–2.62), and 8.15 (95% CI: 7.17–9.27) times higher odds, respectively, of developing any retinal complication. Per diopter increase in myopia, the odds of each complication increased: CNV (OR 1.11; 95% CI: 1.09–1.12), MMD (OR 1.22; 95% CI: 1.18–1.25), foveoschisis (OR 1.22; 95% CI: 1.16–1.28), MH (OR 1.06; 95% CI: 1.05–1.08), FRD (OR 1.23; 95% CI: 1.16–1.32), and RRD (OR 1.10; 95% CI: 1.10–1.11). In severe myopes, odds were markedly elevated: CNV (OR 22.90), MMD (OR 60.19), foveoschisis (OR 102.98), MH (OR 6.69), FRD (OR 22.72), and RRD (OR 6.84).

**Conclusions:**

Myopia is independently associated with higher odds of retinal diseases, and this risk increases incrementally with severity. These findings support a dose-response relationship and highlight the importance of early risk stratification, tailored monitoring, and timely referral in patients with high and severe myopia.

**Supplementary Information:**

The online version contains supplementary material available at 10.1186/s40942-025-00745-7.

## Introduction

By 2050, nearly half of the global population is projected to be diagnosed with myopia [1]. This surge has been attributed to the modification of environmental and lifestyle factors, such as an increased time spent indoors, on electronic devices, and performing near-work activities [2, 3]. Clinically, the ocular complications of myopia can culminate in irreversible vision loss and significantly impair patients’ quality of life. Patients have highlighted the inconvenience of wearing glasses, blurry vision, activity limitations, and low self-esteem as among the most pertinent issues impairing their quality of life [4]. Furthermore, this growing prevalence of myopia globally creates economic burdens as well. In Singapore, the average individual cost for children diagnosed with myopia is approximately $148 per year and rises to $709 per year later into adulthood [5, 6]. Meanwhile, the loss of productivity due to vision loss in myopic patients has been estimated to cost countries between 6.7 and 9.4 billion dollars annually [7]. Ultimately, the disease burden of myopia extends beyond vision impairment by reducing patients’ quality of life, increasing healthcare costs, and lowering productivity.

Axial elongation is the primary driver in the pathophysiology of myopia [8]. This stretching is associated with a decrease in choroidal thickness and retinal pigment epithelium cell layer density while also promoting the development of lacquer cracks and defects in the Bruch’s membrane [8]. Staphylomas are another complication of pathologic myopia characterized by outpouchings in the globe’s contour and can exacerbate the risk of retinal complications in myopic eyes due to the additional stretching they impose on the retinal layers [9]. Consequently, because axial elongation and staphylomas compromise the structural integrity of the retina, myopic eyes are predisposed to ocular complications. Choroidal neovascularization (CNV), myopic macular degeneration (MMD), foveoschisis, macular holes (MH), foveal retinal detachment (FRD), and rhegmatogenous retinal detachments (RRD) are among the retinal sequelae of myopia that can lead to irreversible vision loss if not managed properly [4]. Growing evidence implicates dopamine pathways and the amount of time spent outdoors as critical regulators of these anatomical changes in the sclera and retina [10]. For instance, dopaminergic agents have been shown to be effective in reducing axial elongation and myopic shift in animal models [11, 12]. Meanwhile, other evidence suggests that light from outdoor activities stimulates the synthesis and release of dopamine by retinal cells [10].

The surging prevalence of this refractive disorder despite its economic and disease burden highlights the importance of understanding its pathophysiology and associated complications. Previous studies have explored how each diopter increase in myopia can increase the prevalence of its complications, and so consequently, slowing its progression can lower patients’ risk of other ocular disorders [13, 14]. However, such studies are often restricted either by a relatively small cohort size or focusing their methodology on pediatric patients, limiting the generalizability of results. This epidemiological study is among the first to integrate a large database approach towards exploring the relationship between myopia and its retinal sequelae, drawing on the Stanford University Medical Center Clinical Data Warehouse (STARR) database. By exploring how refractive status, axial length, and spherical equivalents are associated with different ocular complications, we hope to quantify myopia’s relationship with different retinal diseases. Such findings can inspire more personalized risk stratification models and surveillance measures, encouraging better visual outcomes for myopic patients.

## Methods

This research adhered to HIPAA regulations and received approval from the Institutional Review Board (IRB) subcommittees at Stanford Hospital. This retrospective cohort study utilized the STARR database, which contains records for over 5 million pediatric and adult patients at Stanford University dating back to 1995. The database is updated in real-time and incorporates patient data, including demographic information, clinical events, and exam findings such as diagnoses, refractive errors, and axial lengths, from Epic systems implemented at Stanford University since 2008.

We analyzed patients from a secure data repository containing all patients in STARR with at least one documented eye visit and only included those with spherical equivalent or axial length measurements available. Our primary outcomes were retinal sequelae associated with myopia— choroidal neovascularization (CNV), myopic macular degeneration (MMD), foveoschisis, macular hole (MH), rhegmatogenous retinal detachment (RRD), and foveal retinal detachment (FRD). The diagnosis of each sequela was determined by its respective International Classification of Diseases, 10th Revision (ICD-10) code (Supplemental Table 1). Exposure was defined by myopia status, which was determined via spherical equivalents. Patients were classified as either non-myopes (>-0.5 D), myopes (≤-0.5 D to -6 D), high myopes (≤-6 D to -14 D), or severe myopes (≤-14 D). Race and ethnicity data were sourced from STARR. Race was categorized as Asian, American Indian or Alaska Native, Black or African American, Native Hawaiian or Other Pacific Islander, White, Unknown, or Other, while ethnicity was grouped as Hispanic or Latino, Not Hispanic or Latino, or Unknown. Additionally, each patient’s spherical equivalent refraction, retinal diagnoses, as well as the age and laterality at the time of diagnosis were recorded from their most recent visit in the STARR database. Axial length measurements, which are routinely documented in the Myopia Clinic at Stanford’s Byer Eye Institute and integrated into STARR were also obtained. Prevalence tables were constructed to characterize patients without myopia, with myopia, with high myopia, and with severe myopia. These tables were stratified by age (in 10-year bins from 20 to 90+), sex, race, ethnicity, and retinal sequelae. Additionally, we compared the prevalence of the myopic groups using Chi-square tests to assess statistical differences between groups.

We also examined the mean age at diagnosis for each retinal sequela, stratified by myopia status. An ANOVA was used to compare the mean ages across each myopic group. A logistic regression model assessed the likelihood of each retinal sequela, comparing each myopia group to non-myopes. Another model evaluated the association between each of the retinal sequelae and every spherical equivalent diopter increase in myopia. In all models, each retinal sequela was treated as a binary outcome, defined by the presence of the corresponding ICD-10 codes in the patient records. Patients with multiple retinal complications were included as independent measurements in our cohort. Additionally, data from both eyes of each patient were included in our analysis, and this was accounted for by fitting our regression model using generalized estimated equations. Repeat analysis was also performed after categorizing the myopic status of the cohort by axial lengths, where patients were classified as either non-myopes (< 24 mm), myopes (24 mm- <26.5 mm), high myopes (26.5 mm-<30 mm), and severe myopes (≥ 30 mm). By analyzing subgroups of myopia defined by spherical equivalents and axial lengths, we hope to compare the clinical and anatomical definitions of myopia in predicting retinal sequelae.

## Results

Our cohort consisted of 282,810 patients with spherical equivalent measurements and 3,050 patients with axial length measurements available in the STARR database (Table 1, Supplemental Table 2). When stratifying myopic status by spherical equivalents, approximately 79.83% were diagnosed with no myopia, 15.92% with myopia, 3.72% with high myopia, and 0.51% with severe myopia. Patients with CNV, FRD, foveoschisis, MH, MMD, and RRD represented 0.14%, <0.01%, <0.01%, 0.34%, 0.01%, and 1.92% of non-myopes, respectively. Meanwhile, they consisted of 0.85%, 0.01%, 0.04%, 2.18%, 0.10%, and 7.44% of myopes and 1.21%, 0.09%, 0.05%, 2.27%, 0.31%, and 12.92% of high myopes. For severe myopes, they made up 5.95%, 0.42%, 0.70%, 5.67%, 1.89%, and 23.44% of patients. Additionally, the distribution of spherical equivalents among our subgroups of myopia defined by axial lengths was found to be predominantly negative as well (Supplemental Fig. 1), confirming that these eyes were truly myopic.

The mean ages for non-myopes, myopes, high myopes, and severe myopes were 55.29±24.12 years, 47.59±16.97 years, 58.32±20.23 years, and 54.43±19.16 years, respectively. Additionally, patients who identified as female represented 54.05%, 54.07%, 54.79%, and 47.17% of non-myopes, myopes, high myopes, and severe myopes, respectively. This corresponded to a female-to-male sex ratio of approximately 1.18, 1.18, 1.21, and 0.89 for non-myopes, myopes, high myopes, and severe myopes, respectively. Patients who self-identified as White represented the largest proportion of non-myopes (45.74%) and myopes (43.82%), while those identifying as Asian consisted of 40.34% of high myopes and 35.76% of severe myopes. Meanwhile, non-Hispanic patients consisted of the greatest proportion of non-myopes (73.57%), myopes (81.35%), high myopes (84.03%) and severe myopes (77.75%). When comparing these myopic groups, a significant difference was observed after stratifying by age (*p* < 0.001), sex (*p* < 0.001), race (*p* < 0.001), ethnicity (*p* < 0.001), and retinal sequelae (*p* = 0.002). Supplemental Table 2 contains demographic characteristics of the cohort when defining myopic status by axial length.

**Fig. 1:**
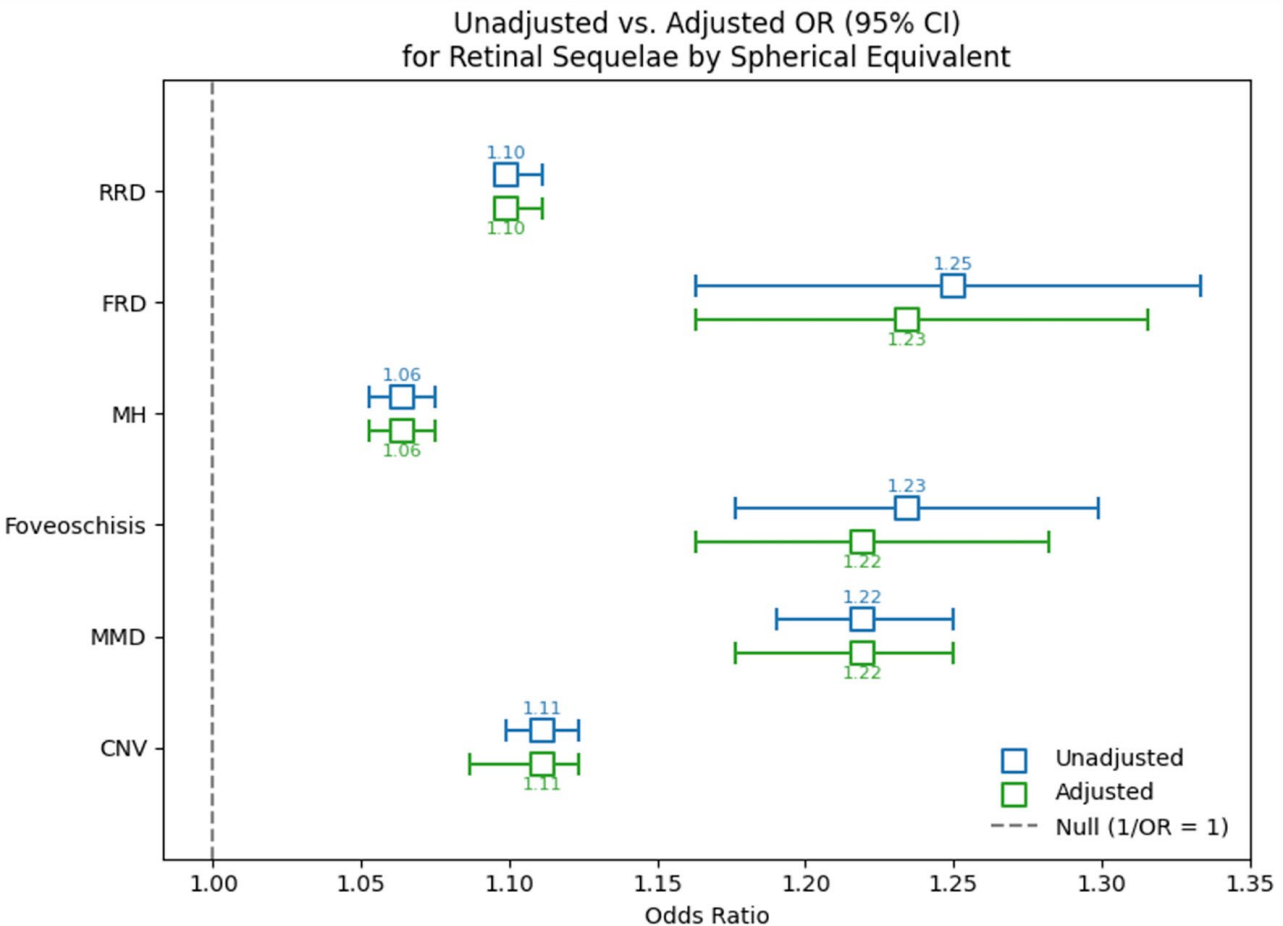
Association between myopic spherical equivalents and each retinal sequelaf

The likelihood of each retinal sequela was found to be associated with myopic status (Table 2). Compared to non-myopes, patients with myopia, high myopia, and severe myopia were associated with a 2.45 (95% CI: 2.36–2.55), 2.46 (95% CI: 2.31–2.62), and 8.15 (95% CI: 7.17–9.27) fold higher likelihood of being diagnosed with any of the retinal sequelae, respectively. Meanwhile, such patients had 2.49 (95% CI: 2.20–2.81), 1.65 (95% CI: 1.34–2.04), and 22.90 (95% CI: 17.19–30.50) times higher odds of being diagnosed with CNV. A diagnosis of MMD was 5.34 (95% CI: 3.70–7.71), 5.72 (95% CI: 3.48–9.42), and 60.19 (95% CI: 32.44-111.69) times more likely in myopes, high myopes, and severe myopes, respectively. A diagnosis of MH was associated with a 2.49 (95% CI: 2.31–2.70), 1.34 (95% CI: 1.16–1.55), 6.69 (95% CI: 5.14–8.70) times higher odds, respectively. A diagnosis of FRD was associated with a 4.72 (95% CI: 1.66–13.43), 40.30 (95% CI: 14.52-111.88), and 22.72 (95% CI: 6.23–82.88) times higher odds, respectively. A diagnosis of RRD was associated with a 2.24 (95% CI: 2.15–2.34), 2.66 (95% CI: 2.49–2.84), and 6.84 (95% CI: 5.94–7.87) higher odds, respectively. Lastly, a diagnosis of foveoschisis was associated with a 10.95 (95% CI: 5.81–20.64) and 102.98 (95% CI: 35.43-299.27) higher odds among myopes and severe myopes. Supplemental Table 3 outlines these results when categorizing myopic status by axial lengths. Additionally, a significant relationship was also observed between each retinal sequelae and spherical equivalents (Fig. 1). Every diopter increase in myopia was associated with a 1.11 (95% CI: 1.09–1.12), 1.22 (95% CI: 1.18–1.25), 1.22 (95% CI: 1.16–1.28), 1.06 (95% CI: 1.05–1.08), 1.23 (95% CI: 1.16–1.32), and 1.10 (95% CI: 1.10–1.11) times higher likelihood of developing CNV, MMD, foveoschisis, MH, FRD, and RRD, respectively. Supplemental Fig. 2 demonstrates the association between each sequela and increasing axial lengths.

**Fig. 2:**
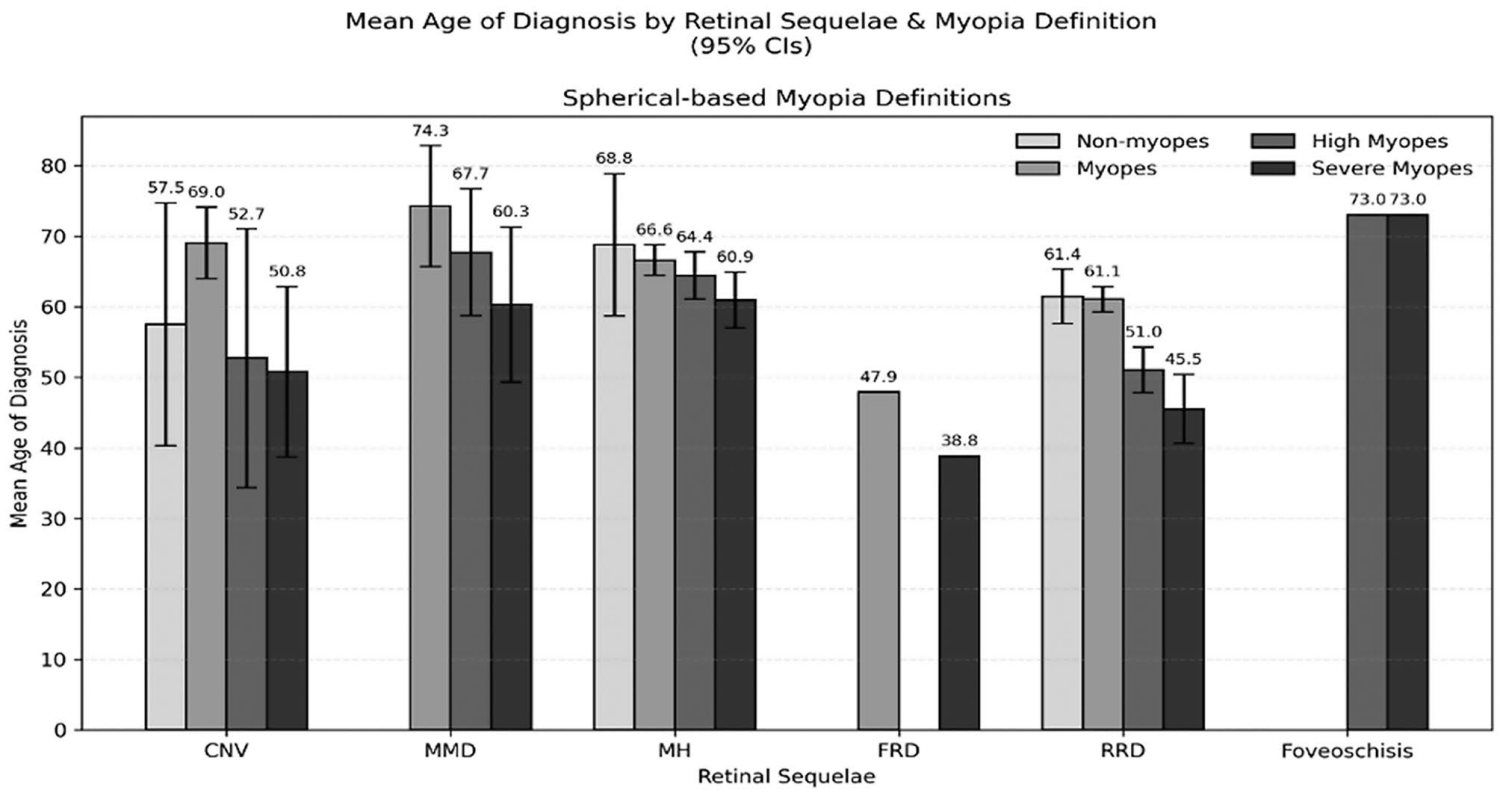
Mean age of diagnosis for retinal sequelae stratified by myopia status determined by spherical equivalents

Figure 2 reports the mean age for the diagnoses of each of the retinal sequelae. On average, CNV was diagnosed at 57.5 ± 17.2, 69.0 ± 5.1, 52.7 ± 18.3, and 50.8 ± 12.1 years for non-myopes, myopes, high myopes, and severe myopes, respectively (*p* = 0.02). Similarly, MH at 68.8 ± 10.1, 66.6 ± 2.2, 64.4 ± 3.4, and 60.9 ± 4.0 years (*p* = 0.58), while RRD was diagnosed at 61.4 ± 3.9, 61.1 ± 1.8, 51.0 ± 3.2, and 45.5 ± 4.9 years (*p* > 0.0001), respectively. MMD was diagnosed at 74.3 ± 8.6, 67.7 ± 9.0, and 60.3 ± 11.0 years for myopes, high myopes, and severe myopes (*p* = 0.24). FRD was diagnosed at 47.9 ± 0.0 and 38.8 ± 0.0 years for myopes and severe myopes (p = NA), while foveoschisis was diagnosed at 73.0 ± 0.0 and 73.0 ± 0.0 years for high myopes and severe myopes (p = NA). Supplemental Fig. 3 demonstrates these results when categorizing myopic status by axial lengths.

## Discussion

The growing prevalence of myopia represents a global health crisis that threatens the sight of patients. Furthermore, its associated complications carry a socioeconomic burden as well by reducing patients’ quality of life, increasing their costs for healthcare, and lowering productivity [4–7]. Thus, the growing disease burden of myopia warrants exploration of how it contributes to retinal diseases. This study demonstrated that myopia is associated with a higher likelihood of the retinal sequelae examined. By stratifying this association according to patients’ myopic status and spherical equivalent, we highlight how the strength of this predisposition towards retinal diseases increases with higher degrees of myopia as well. Patients with severe myopia were also found to be diagnosed with these retinal diseases earlier, reaffirming the positive association between the severity of myopia and the likelihood of these ocular complications. Lastly, both spherical equivalents and axial lengths had similar associations with each retinal sequelae, supporting the robustness of either definition of myopia in predicting the complications of myopia.

We observed a significant difference in the prevalence of non-myopia, myopia, high myopia, and severe myopia when stratifying our cohort by age, sex, race, and ethnicity. These demographic variables are known contributors to the epidemiology of myopia. Younger age is associated with faster progression of myopia and predisposes patients to more severe manifestations in the future [15]. One study found that female patients not only were 1.81 times more likely to develop myopia than males but also to progress faster, at a rate of approximately 0.02 D per year [16]. Similarly, another reported Asian children to have comparable progression rates in myopia with White children, but significantly faster rates than Hispanic, Black, and Native American children [17]. Our findings reaffirm these reports for a larger U.S. based population.

Myopia was also observed to increase the likelihood of CNV, MMD, foveoschisis, MH, FRD, and RRD. Axial elongation in myopic eyes induces structural changes to the retina that compromise its integrity, implicating myopia in the development of these ocular complications. Seko et al., for example, demonstrated how the stretching of the retinal pigment epithelial layer can lead to the upregulation of vascular endothelial growth factor, offering a mechanistic model for the development of CNV in myopic eyes [18]. Choroidal thinning in elongated eyes has also been proposed to lead to localized ischemia with subsequent upregulation of angiogenic factors, leading to CNV as well [19]. Meanwhile, axial elongation can also exert tractional forces on the retinal layers that promote the development of MH and RRD [20]. The mechanical stress associated with it can also create posterior staphylomas along the posterior pole of the eye. These distortions of the scleral wall, along with the development of lacquer cracks and chorioretinal atrophy, weaken the macula and encourage CNV, foveoschisis, FRD, and MMD [4, 21, 22]. This study supports such pathophysiologic explanations by substantiating how higher degrees of myopia and longer axial lengths can incrementally increase the odds of these retinal complications, corroborating the role of myopia in each pathophysiology.

Prior studies have also echoed our results. In their systematic review, Wong et al., measured the incidence rate of CNV in eyes with pathologic myopic to be approximately 10.2% over a 10-year follow-up period [23]. Our results similarly highlight the association between CNV and myopia, while also quantifying the incremental growth in the relationship with higher myopic spherical equivalents. Meanwhile, Haarman et al., similarly demonstrated how the likelihood of retinal complications increases with increasing degrees of myopia. They observed low, moderate, and high myopes to be associated with a 13.57-, 72.74-, and 845.08-fold higher likelihood of MMD and a 3.15-, 8.74-, and 12.62-fold higher likelihood of RRD, respectively [24]. Lastly, Kobayashi et al., observed a positive correlation between the age of onset of MH and both the severity of myopia (*R* = 0.689) and axial length (*R* = 0.723) [25]. As with these prior studies, our findings support how myopia predisposes patients to ocular complications. However, our analysis pushes further by detailing the incremental changes in these relationships with varying myopic statuses, axial lengths, and spherical equivalents.

Ultimately, as the global prevalence of myopia rises, this study offers a more nuanced understanding of the retinal complications facing myopic patients. Our findings can help inform risk stratification models when evaluating such patients, encouraging the earlier detection and management of these diseases. For instance, such knowledge can support the identification of high-risk patients in need of more regular surveillance and support more informed patient counseling. During eye examinations, it can also cue physicians and patients on which exam findings and symptoms should flag their concern for these retinal complications as well, encouraging reshaping referrals to retina specialists and improving the prognostication of pathologic myopia. With earlier detection and more proactive monitoring, the visual outcomes of myopic patients may improve.

This study is among the largest cohort studies evaluating the retinal sequelae of myopia in the United States. The STARR database contains records of over 5 million patients, which helps to increase the generalizability of our results while minimizing the effects of confounders. However, despite this, patients from marginalized communities are underrepresented within the STARR database, potentially limiting the generalizability of our results to broader populations. Patients with high myopia, severe myopia, MMD, foveoschisis, and FRD were disproportionately underrepresented within our cohort. This may have exaggerated our findings about these retinal sequelae and may also explain the insignificant results from our logistic regression. This may also explain why, contrary to the current literature and supported by our wide confidence intervals, high myopes were found to be less likely to develop CNV than myopes but more likely to develop FRD than severe myopes [26, 27]. Similarly, while axial lengths may offer accurate representations of myopic status, the disproportionately smaller cohort size when classifying myopia by axial lengths may explain why weaker associations between myopia and its sequelae were observed in our supplementary analysis [28]. Lastly, our analysis did not exclude non-myopic patients with retinal complications secondary to other ocular disorders or account for postoperative refractive changes following cataract surgery. This may have introduced misclassification bias in our analysis but is partially mitigated by our supplementary analysis relying on axial lengths, which reported concordant findings as our primary results. Ultimately, future studies that circumvent these restrictions by including more representative and diverse patient cohorts can improve how well such findings can be extrapolated onto larger populations. Similarly, incorporating broader clinical data, such as results from eye exams or OCT imaging, can offer a more granular understanding of myopia’s involvement in the pathophysiology of these retinal diseases. 

Overall, this study offers epidemiological insights into the relationship between myopia and different retinal diseases. We demonstrated how myopia predisposes patients to CNV, MMD, foveoschisis, MH, FRD, and RRD. Furthermore, our results stratified this association by patients’ myopic status and spherical equivalents. In doing so, we hope to offer a model for risk stratification when evaluating myopic patients to improve patient prognoses. With more proactive management of these patients, we hope to improve patient counseling and visual outcomes, reducing the disease burden of myopia.

**Table 1:** Baseline characteristics of patients in STARR by myopia status as determined by spherical equivalents

Characteristic	Patients, No. (%)				*P* value
	Non-Myopes	Myopes	High Myopes	Severe Myopes	
	*n* = 225,797	*n* = 45,038	*n* = 10,546	*n* = 1429	
**Age**,** y**					
20–29	14,940 (6.62%)	2794 (6.2%)	844 (8.0%)	131 (9.17%)	< 0.001
30–39	28,309 (12.54%)	7181 (15.94%)	2630 (24.94%)	153 (10.71%)	
40–49	31,282 (13.85%)	5129 (11.39%)	1668 (15.82%)	173 (12.11%)	
50–59	30,562 (13.54%)	5069 (11.25%)	1391 (13.19%)	250 (17.49%)	
60–69	33,475 (14.83%)	6694 (14.86%)	1666 (15.8%)	319 (22.32%)	
70–79	31,454 (13.93%)	8522 (18.92%)	1427 (13.53%)	243 (17.0%)	
80–89	19,732 (8.74%)	6252 (13.88%)	597 (5.66%)	71 (4.97%)	
≥ 90	12,047 (5.34%)	2966 (6.59%)	181 (1.72%)	27 (1.89%)	
Unknown	23,996 (10.63%)	431 (0.96%)	142 (1.35%)	62 (4.34%)	
**Sex**					
Male	103,720 (45.94%)	20,681 (45.92%)	4765 (45.18%)	755 (52.83%)	< 0.001
Female	122,036 (54.05%)	24,354 (54.07%)	5778 (54.79%)	674 (47.17%)	
Unknown	41 (0.02%)	3 (0.01%)	3 (0.03%)	0 (0.0%)	
**Race**					
White	103,290 (45.74%)	19,734 (43.82%)	3488 (33.07%)	445 (31.14%)	< 0.001
Unknown	24,447 (10.83%)	3042 (6.75%)	831 (7.88%)	71 (4.97%)	
Asian	43,703 (19.35%)	11,515 (25.57%)	4254 (40.34%)	511 (35.76%)	
Black or African American	8980 (3.98%)	1399 (3.11%)	221 (2.1%)	34 (2.38%)	
American Indian or Alaska Native	704 (0.31%)	130 (0.29%)	30 (0.28%)	1 (0.07%)	
Native Hawaiian or Other Pacific Islander	2378 (1.05%)	391 (0.87%)	74 (0.7%)	28 (1.96%)	
Other race	42,295 (18.73%)	8827 (19.6%)	1648 (15.63%)	339 (23.72%)	
**Ethnicity**					
Hispanic or Latino	32,543 (14.41%)	5557 (12.34%)	906 (8.59%)	248 (17.35%)	< 0.001
Not Hispanic or Latino	166,116 (73.57%)	36,638 (81.35%)	8862 (84.03%)	1111 (77.75%)	
Unknown	27,138 (12.02%)	2843 (6.31%)	778 (7.38%)	70 (4.9%)	
**Retinal Sequelae**					
CNV	308 (0.14%)	384 (0.85%)	128 (1.21%)	85 (5.95%)	< 0.001
FRD	7 (<0.01%)	5 (0.01%)	10 (0.09%)	6 (0.42%)	
Foveoschisis	6 (<0.01%)	16 (0.04%)	5 (0.05%)	10 (0.70%)	
MH	773 (0.34%)	983 (2.18%)	239 (2.27%)	81 (5.67%)	
MMD	12 (0.01%)	46 (0.10%)	33 (0.31%)	27 (1.89%)	
RRD	4341 (1.92%)	3349 (7.44%)	1363 (12.92%)	335 (23.44%)	

CNV: choroidal neovascularization; FRD: foveal retinal detachment; MH: macular hole; MMD: myopic macular degeneration; RRD: rhegmatogenous retinal detachment

**Table 2:** Odds of retinal sequelae stratified by myopia status determined by spherical equivalents

Retina sequelae	Myopia severity	Odds ratio (95% CI)*	99% CI
Any	Non-Myope	1 (Reference)	Reference
	Myope	2.45 (2.36–2.55)	2.33–2.58
	High Myope	2.46 (2.31–2.62)	2.27–2.67
	Severe Myope	8.15 (7.17–9.27)	6.88–9.65
CNV	Non-Myope	1 (Reference)	Reference
	Myope	2.49 (2.20–2.81)	2.12–2.92
	High Myope	1.65 (1.34–2.04)	1.26–2.17
	Severe Myope	22.90 (17.19–30.50)	15.71–33.38
MMD	Non-Myope	1 (Reference)	Reference
	Myope	5.34 (3.70–7.71)	3.29–8.65
	High Myope	5.72 (3.48–9.42)	2.97–11.02
	Severe Myope	60.19 (32.44–111.69)	26.71–135.63
Foveoschisis	Non-Myope	1 (Reference)	Reference
	Myope	10.95 (5.81–20.64)	4.76–25.19
	High Myope	1.59 (0.46–5.49)	0.31–8.11
	Severe Myope	102.98 (35.43–299.27)	25.34–418.47
MH	Non-Myope	1 (Reference)	Reference
	Myope	2.49 (2.31–2.70)	2.25–2.76
	High Myope	1.34 (1.16–1.55)	1.11–1.62
	Severe Myope	6.69 (5.14–8.70)	4.73–9.45
FRD	Non-Myope	1 (Reference)	Reference
	Myope	4.72 (1.66–13.43)	1.19–18.65
	High Myope	40.30 (14.52–111.88)	10.53–154.20
	Severe Myope	22.72 (6.23–82.88)	4.15–124.46
RRD	Non-Myope	1 (Reference)	Reference
	Myope	2.24 (2.15–2.34)	2.12–2.38
	High Myope	2.66 (2.49–2.84)	2.44–2.90
	Severe Myope	6.84 (5.94–7.87)	5.68–8.23

CNV: choroidal neovascularization; FRD: foveal retinal detachment; MH: macular hole; MMD: myopic macular degeneration; RRD: rhegmatogenous retinal detachment

* Models examine odds of MMD, myopic traction maculopathy (foveoschisis, MH, FRD), and retinal detachments in patients with and without varying levels of myopia. Models adjusted for age, sex, race, and ethnicity

## Supplementary Information

Below is the link to the electronic supplementary material.

Supplementary Material 1

## Data Availability

The datasets used and/or analysed during the current study are available from the corresponding author on reasonable request.

## References

[1] Liang J. Trend and projection of myopia in children and adolescents from 1990 to 2050: A comprehensive systematic review and Meta-analysis. Br J Ophthalmol. 2025;362:109. 10.1136/bjo-2024-325427 39317432

[2] Morgan IG, French AN, Ashby RS, Guo X, Ding X, He M, et al. The epidemics of myopia: aetiology and prevention. Prog Retin Eye Res. 2018;62:134–49. 28951126 10.1016/j.preteyeres.2017.09.004

[3] Tsai T-H, Liu Y-L, Ma I-H, Su C-C, Lin C-W, Lin LL-K, et al. Evolution of the prevalence of myopia among Taiwanese schoolchildren: a review of survey data from 1983 through 2017. Ophthalmology. 2021;128(2):290–301. 32679159 10.1016/j.ophtha.2020.07.017

[4] Sankaridurg P, Tahhan N, Kandel H, Naduvilath T, Zou H, Frick KD, et al. IMI impact of myopia. Investig Ophthalmol Vis Sci. 2021;62(5):2. 10.1167/iovs.62.5.2 PMC8083082 33909036

[5] Lim M, Gazzard G, Sim E, Tong L, Saw S. Direct costs of myopia in Singapore. Eye. 2009;23(5):1086–9. 18670466 10.1038/eye.2008.225

[6] Zheng Y-F, Pan C-W, Chay J, Wong TY, Finkelstein E, Saw S-M. The economic cost of myopia in adults aged over 40 years in Singapore. Investig Ophthalmol Vis Sci. 2013;54(12):7532–7. 24159089 10.1167/iovs.13-12795

[7] Ma Y, Wen Y, Zhong H, Lin S, Liang L, Yang Y, et al. Healthcare utilization and economic burden of myopia in urban China: a nationwide cost-of-illness study. J Global Health. 2022;12:11003. 10.7189/jogh.12.11003 PMC8934110 35356656

[8] Jonas JB, Jonas RA, Bikbov MM, Wang YX, Panda-Jonas S. Myopia: histology, clinical features, and potential implications for the etiology of axial elongation. Prog Retin Eye Res. 2023;96:101156. 36585290 10.1016/j.preteyeres.2022.101156

[9] Ohno-Matsui K, Jonas JB. Posterior Staphyloma in pathologic myopia. Prog Retin Eye Res. 2019;70:99–109. 30537538 10.1016/j.preteyeres.2018.12.001

[10] Zhang J, Deng G. Protective effects of increased outdoor time against myopia: a review. J Int Med Res. 2020;48(3):0300060519893866. 31854216 10.1177/0300060519893866 PMC7607527

[11] Junfeng M, Shuangzhen L, Wenjuan Q, Fengyun L, Xiaoying W, Qian T. Levodopa inhibits the development of form-deprivation myopia in Guinea pigs. Optom Vis Sci. 2010;87(1):53–60. 19901858 10.1097/OPX.0b013e3181c12b3d

[12] Gao Q, Liu Q, Ma P, Zhong X, Wu J, Ge J. Effects of direct intravitreal dopamine injections on the development of lid-suture induced myopia in rabbits. Graefe’s Archive Clin Experimental Ophthalmol. 2006;244(10):1329–35. 10.1007/s00417-006-0254-1 16550409

[13] Bullimore MA, Brennan NA. Myopia control: why each diopter matters. Optom Vis Sci. 2019;96(6):463–5. 31116165 10.1097/OPX.0000000000001367

[14] Bullimore MA, Ritchey ER, Shah S, Leveziel N, Bourne RR, Flitcroft DI. The risks and benefits of myopia control. Ophthalmology. 2021;128(11):1561–79. 33961969 10.1016/j.ophtha.2021.04.032

[15] Gwiazda J, Hyman L, Dong LM, Everett D, Norton T, Kurtz D, et al. Factors associated with high myopia after 7 years of follow-up in the correction of myopia evaluation trial (COMET) cohort. Ophthalmic Epidemiol. 2007;14(4):230–7. 17896302 10.1080/01658100701486459

[16] Lee SS-Y, Lingham G, Sanfilippo PG, Hammond CJ, Saw S-M, Guggenheim JA, et al. Incidence and progression of myopia in early adulthood. JAMA Ophthalmol. 2022;140(2):162–9. 34989764 10.1001/jamaophthalmol.2021.5067 PMC8739830

[17] Jones-Jordan LA, Sinnott LT, Chu RH, Cotter SA, Kleinstein RN, Manny RE, et al. Myopia progression as a function of sex, age, and ethnicity. Investig Ophthalmol Vis Sci. 2021;62(10):36. 10.1167/iovs.62.10.36 PMC8411866 34463720

[18] Seko Y, Seko Y, Fujikura H, Pang J, Tokoro T, Shimokawa H. Induction of vascular endothelial growth factor after application of mechanical stress to retinal pigment epithelium of the rat in vitro. Investig Ophthalmol Vis Sci. 1999;40(13):3287–91. 10586955

[19] Wakabayashi T, Ikuno Y. Choroidal filling delay in choroidal neovascularisation due to pathological myopia. Br J Ophthalmol. 2010;94(5):611–5. 19846414 10.1136/bjo.2009.163535

[20] Stirpe M, Michels RG. Retinal detachment in highly myopic eyes due to macular holes and epiretinal traction. Retina. 1990;10(2):113–4. 2402551 10.1097/00006982-199004000-00004

[21] Takano M, Kishi S. Foveal retinoschisis and retinal detachment in severely myopic eyes with posterior Staphyloma. Am J Ophthalmol. 1999;128(4):472–6. 10577588 10.1016/s0002-9394(99)00186-5

[22] Kim Y, Yoon J, Koh H. The analysis of lacquer crack in the assessment of myopic choroidal neovascularization. Eye. 2011;25(7):937–46. 21527958 10.1038/eye.2011.94 PMC3178161

[23] Wong TY, Ferreira A, Hughes R, Carter G, Mitchell P. Epidemiology and disease burden of pathologic myopia and myopic choroidal neovascularization: an evidence-based systematic review. Am J Ophthalmol. 2014;157(1):9–25. e12. 24099276 10.1016/j.ajo.2013.08.010

[24] Haarman AE, Enthoven CA, Tideman JWL, Tedja MS, Verhoeven VJ, Klaver CC. The complications of myopia: a review and meta-analysis. Investig Ophthalmol Vis Sci. 2020;61(4):49. 10.1167/iovs.61.4.49 PMC7401976 32347918

[25] Kobayashi H, Kobayashi K, Okinami S. Macular hole and myopic refraction. Br J Ophthalmol. 2002;86(11):1269–73. 12386087 10.1136/bjo.86.11.1269 PMC1771378

[26] Wu P, Chen Y, Chen Y-H, Chen C, Shin S, Tsai C, et al. Factors associated with foveoschisis and foveal detachment without macular hole in high myopia. Eye. 2009;23(2):356–61. 18064059 10.1038/sj.eye.6703038

[27] Young TL, Metlapally R, Shay AE. Complex trait genetics of refractive error. Arch Ophthalmol. 2007;125(1):38–48. 17210850 10.1001/archopht.125.1.38

[28] Tideman JWL, Snabel MC, Tedja MS, Van Rijn GA, Wong KT, Kuijpers RW, et al. Association of axial length with risk of uncorrectable visual impairment for Europeans with myopia. JAMA Ophthalmol. 2016;134(12):1355–63. 27768171 10.1001/jamaophthalmol.2016.4009

