# Supplementary material for: Beyond refractive error: myopia’s exponential burden on retinal health with each diopter
Source: Int J Retina Vitreous. 2025 Nov 4;11:121. doi: 10.1186/s40942-025-00745-7 (PMC12584467; doi:10.1186/s40942-025-00745-7)

**Supplemental Table 1.** The CPT and ICD-10 codes utilized for determining patients with outpatient visits and a diagnoses of each retinal sequela

| **Disease/Procedure** | **Classification** | **Code** |
| --- | --- | --- |
| Outpatient Eye Visit | CPT | 99202, 99203, 99204, 99205, 92002, 92004, 99211, 99212, 99213, 99214, 99215, 92012, 92014, 99024 |
| Choroidal Neovascularization | ICD-10 | H44.2A*, H35.05* |
| Macular Hole | ICD-10 | H44.2B*, H35.34* |
| Rhegmatogenous Retinal Detachment | ICD-10 | H33.0* |
| Foveal Retinal Detachment | ICD-10 | H44.2C* |
| Foveoschisis | ICD-10 | H44.2D* |
| Primary Open Angle Glaucoma | ICD-10 | H40.11* |
| Glaucoma Suspect | ICD-10 | H40.0* |

|  | **Patients, No. (%)** | | | |  |
| --- | --- | --- | --- | --- | --- |
| **Characteristic** | **Non-Myopes** | **Myopes** | **High Myopes** | **Severe Myopes** | ***P* value** |
|  | **n = 1412** | **n = 1198** | **n = 376** | **n = 64** |  |
| **Age, y** |  |  |  |  |  |
| 20 - 29 | 12 (0.85) | 17 (1.42) | 10 (2.66) | 1 (1.56) | <0.001 |
| 30 - 39 | 12 (0.85) | 21 (1.75) | 14 (3.72) | 5 (7.81) |  |
| 40 - 49 | 24 (1.7) | 38 (3.17) | 15 (3.99) | 5 (7.81) |  |
| 50 - 59 | 87 (6.16) | 91 (7.6) | 54 (14.36) | 8 (12.5) |  |
| 60 - 69 | 249 (17.63) | 279 (23.29) | 119 (31.65) | 19 (29.69) |  |
| 70 - 79 | 491 (34.77) | 464 (38.73) | 111 (29.52) | 23 (35.94) |  |
| 80 - 89 | 412 (29.18) | 219 (18.28) | 34 (9.04) | 2 (3.13) |  |
| ≥90 | 125 (8.85) | 55 (4.59) | 6 (1.6) | 1 (1.56) |  |
| Unknown | 0 (0) | 14 (1.17) | 13 (3.46) | 0 (0) |  |
| **Sex** |  |  |  |  |  |
| Male | 489 (34.63) | 692 (57.76) | 214 (56.91) | 29 (45.31) | <0.001 |
| Female | 923 (65.37) | 506 (42.24) | 162 (43.09) | 35 (54.69) |  |
| Unknown | 0 (0) | 0 (0) | 0 (0) | 0 (0) |  |
| **Race** |  |  |  |  |  |
| White | 468 (33.14) | 529 (44.16) | 127 (33.78) | 15 (23.44) | <0.001 |
| Unknown | 16 (1.13) | 18 (1.5) | 9 (2.39) | 1 (1.56) |  |
| Asian | 367 (25.99) | 327 (27.3) | 169 (44.95) | 32 (50) |  |
| Black or African | 32 (2.27) | 33 (2.75) | 7 (1.86) | 0 (0) |  |
| American Indians | 4 (0.28) | 5 (0.42) | 0 (0) | 0 (0) |  |
| Native Hawaiians | 21 (1.49) | 15 (1.25) | 0 (0) | 0 (0) |  |
| Other race | 504 (35.69) | 271 (22.62) | 64 (17.02) | 16 (25) |  |
| **Ethnicity** |  |  |  |  |  |
| Hispanic or Latino | 312 (22.1) | 172 (14.36) | 28 (7.45) | 12 (18.75) | <0.001 |
| Not Hispanic or Latino | 1081 (76.56) | 1010 (84.31) | 336 (89.36) | 51 (79.69) |  |
| Unknown | 19 (1.35) | 16 (1.34) | 12 (3.19) | 1 (1.56) |  |
| **Sequelae** |  |  |  |  |  |
| CNV | 13 (0.92) | 20 (1.67) | 7 (1.86) | 7 (10.94) | 0.002 |
| FRD | 0 (0) | 0 (0) | 2 (0.53) | 0 (0) |  |
| Foveoschisis | 0 (0) | 0 (0) | 1 (0.27) | 0 (0) |  |
| MH | 46 (3.26) | 51 (4.26) | 34 (9.04) | 15 (23.44) |  |
| MMD | 0 (0) | 3 (0.25) | 7 (1.86) | 4 (6.25) |  |
| RRD | 99 (7.01) | 166 (13.86) | 116 (30.85) | 23 (35.94) |  |

**CNV**: choroidal neovascularization; **FRD**: foveal retinal detachment; **MH**: macular hole; **MMD**: myopic macular degeneration; **RRD**: rhegmatogenous retinal detachment

**Supplemental Table 2.** Baseline Characteristics of Patients in STARR by Myopic Status as Determined by Axial Lengths

| **Retina Sequelae** | **Myopia Severity** | **Odds ratio (95% CI)*** | **99% CI** |
| --- | --- | --- | --- |
| Any | Non-Myope | 1 (Reference) | Reference |
|  | Myope | 1.25 (1.01 - 1.53) | 0.95 - 1.63 |
|  | High Myope | 2.99 (2.33 - 3.84) | 2.15 - 4.15 |
|  | Severe Myope | 5.01 (2.94 - 8.51) | 2.49 - 10.06 |
| CNV | Non-Myope | 1 (Reference) | Reference |
|  | Myope | 0.91 (0.51 - 1.63) | 0.42 - 1.96 |
|  | High Myope | 0.46 (0.20 - 1.08) | 0.15 - 1.41 |
|  | Severe Myope | 6.95 (2.77 - 17.41) | 2.08 - 23.24 |
| MMD | Non-Myope | 1 (Reference) | Reference |
|  | Myope | 0.31 (0.09 - 1.07) | 0.06 - 1.58 |
|  | High Myope | 1.51 (0.56 - 4.06) | 0.41 - 5.54 |
|  | Severe Myope | 5.73 (1.58 - 20.69) | 1.06 - 30.99 |
| Foveoschisis | Non-Myope | 1 (Reference) | Reference |
|  | Myope | did not converge | did not converge |
|  | High Myope | did not converge | did not converge |
|  | Severe Myope | did not converge | did not converge |
| MH | Non-Myope | 1 (Reference) | Reference |
|  | Myope | 0.74 (0.52 - 1.05) | 0.47 - 1.18 |
|  | High Myope | 1.30 (0.86 - 1.96) | 0.75 - 2.23 |
|  | Severe Myope | 4.35 (2.29 - 8.25) | 1.88 - 10.09 |
| FRD | Non-Myope | 1 (Reference) | Reference |
|  | Myope | 0.00 (0.00 - inf) | 0.00 - inf |
|  | High Myope | 1.14 (0.13 - 10.32) | 0.06 - 20.62 |
|  | Severe Myope | 0.00 (0.00 - inf) | 0.00 - inf |
| RRD | Non-Myope | 1 (Reference) | Reference |
|  | Myope | 1.32 (1.05 - 1.67) | 0.97 - 1.80 |
|  | High Myope | 3.16 (2.42 - 4.14) | 2.22 - 4.50 |
|  | Severe Myope | 3.22 (1.83 - 5.65) | 1.53 - 6.75 |

**CNV**: choroidal neovascularization; **FRD**: foveal retinal detachment; **MH**: macular hole; **MMD**: myopic macular degeneration; **RRD**: rhegmatogenous retinal detachment

* Models examine odds of MMD, MTM, and retinal detachments in patients with and without varying levels of myopia. Models adjusted for age, sex, race, and ethnicity.

**Supplemental Table 3.** Odds of Myopic Macular Degeneration (MMD), Myopic Traction Maculopathy (MTM), and Retinal Detachments by Myopia Status determined by Axial Length

**Supplemental Figure 1**. Distribution of spherical equivalents for the cohort of patients in which myopia was defined by axial length


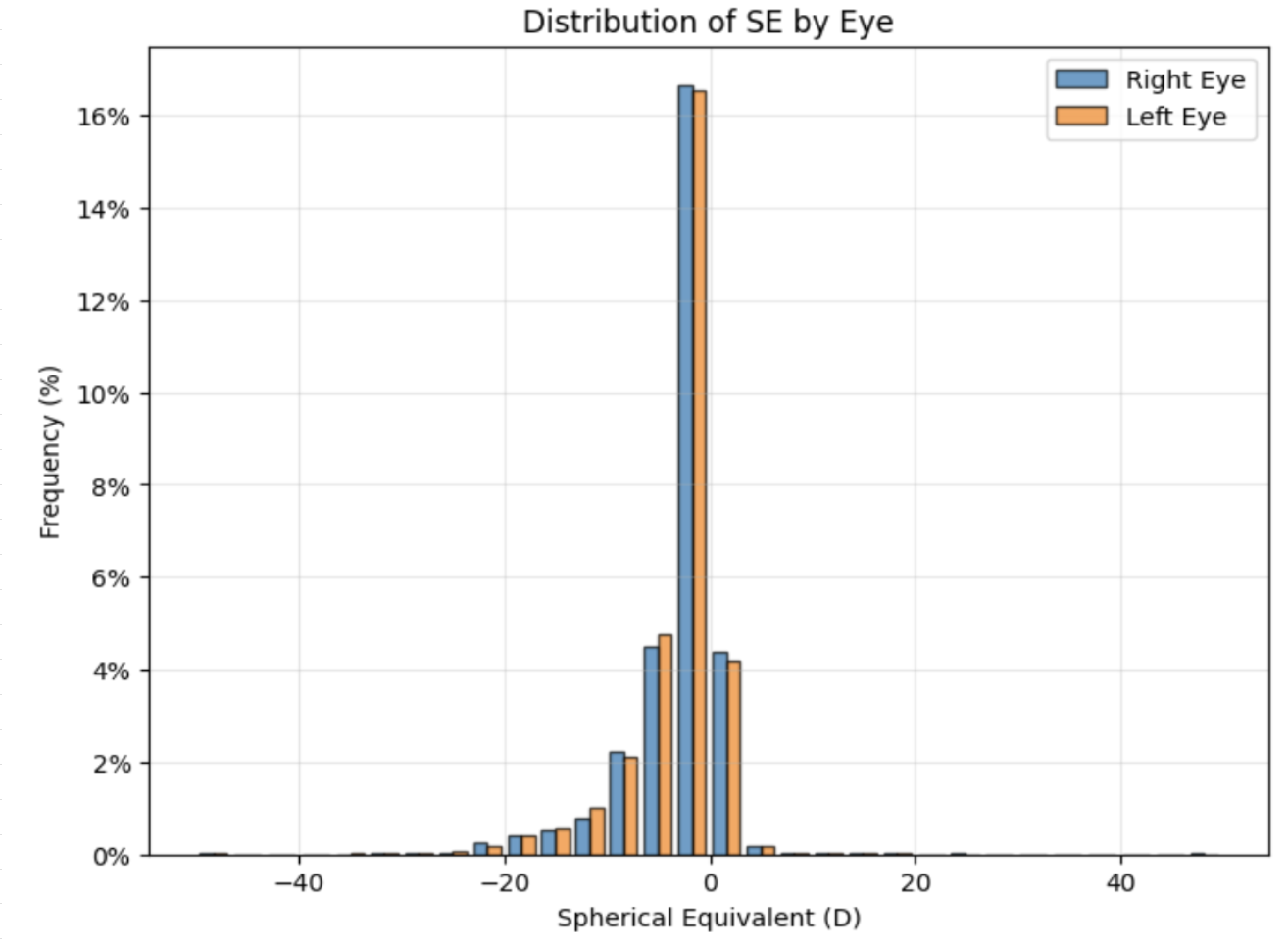


**Supplemental Figure 2.** Association between axial length and each retinal sequela


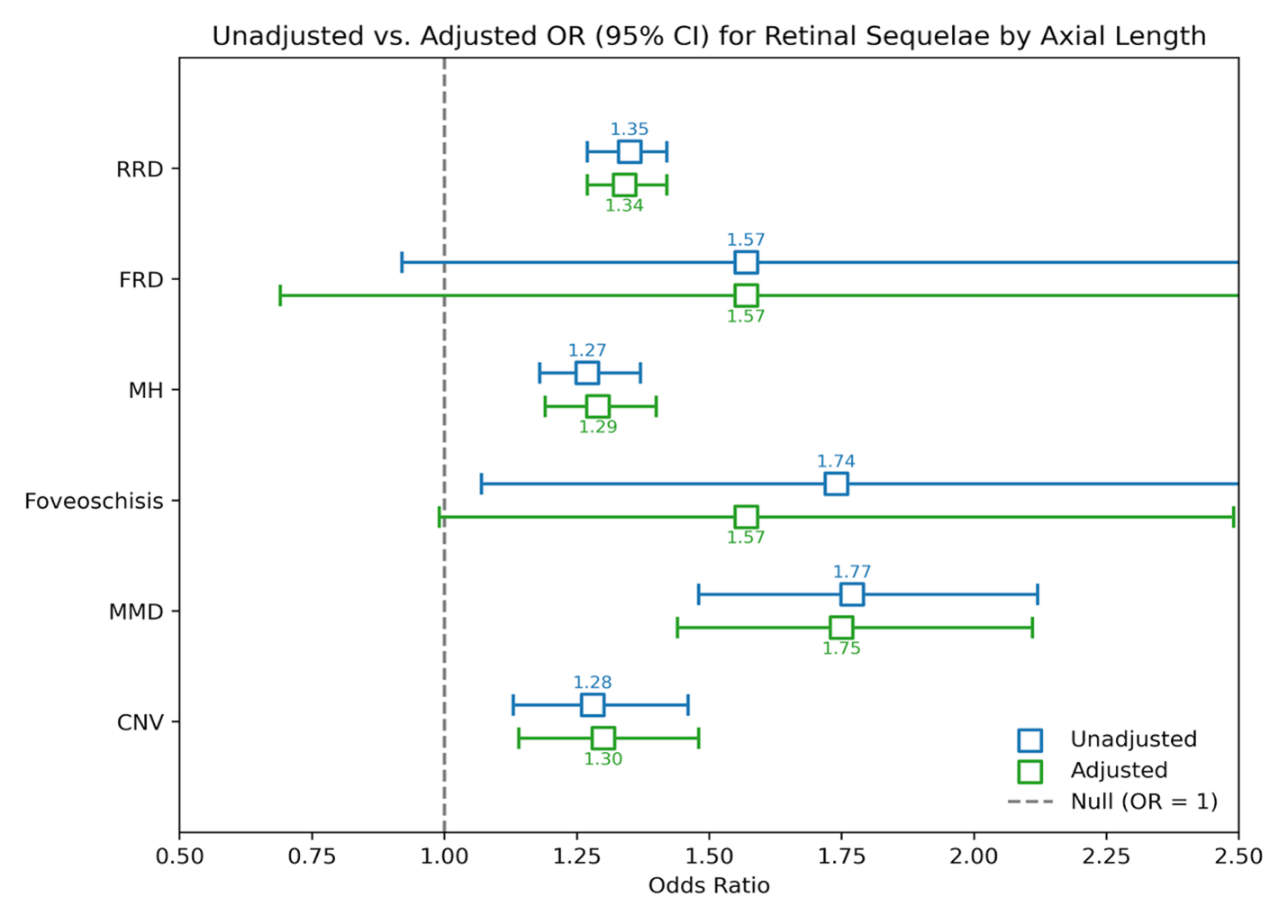


**Supplemental Figure 3.** Mean age of diagnosis for retina sequelae stratified by myopia status determined by axial lengths


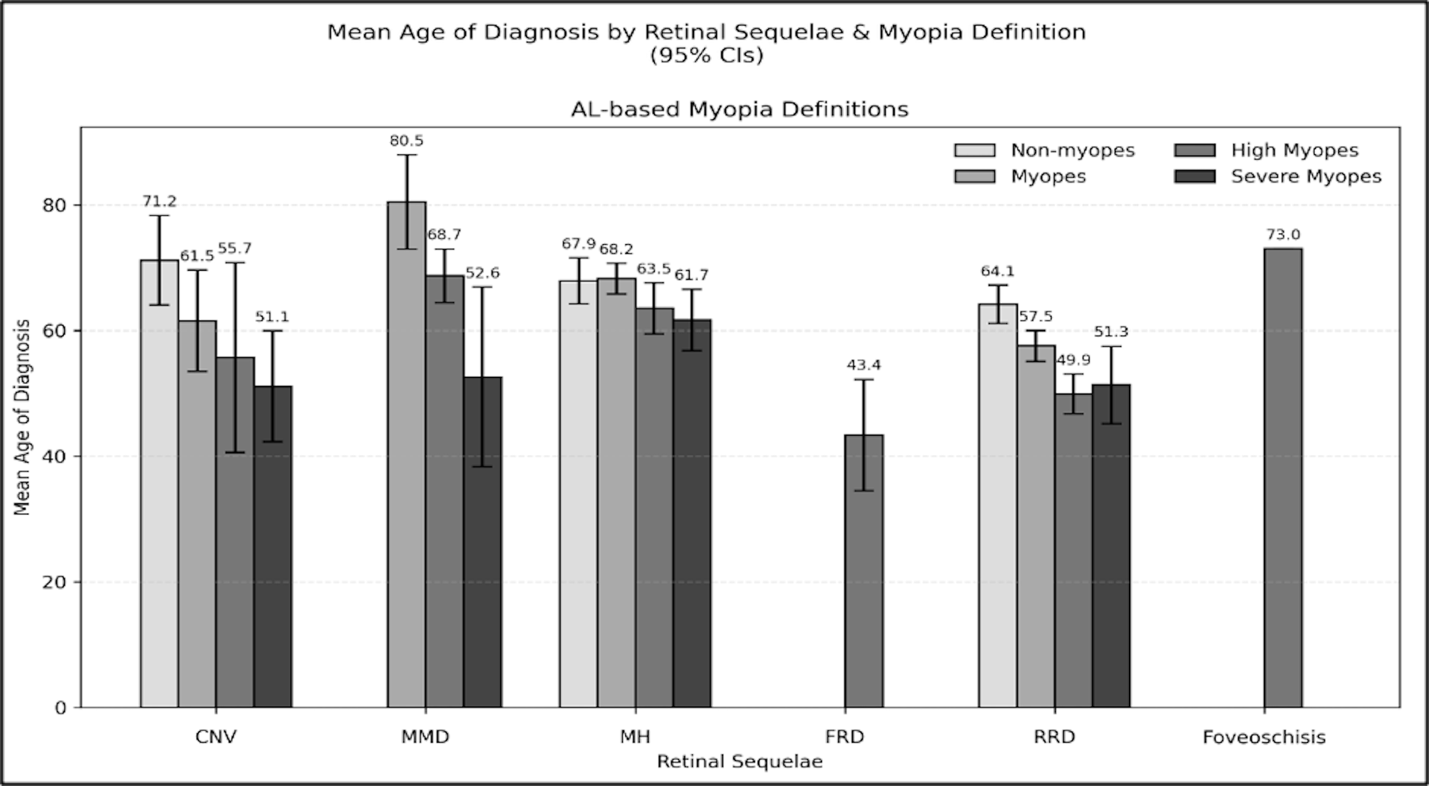

Supplement: Supplementary file 1 — Supplementary Material 1 [file 40942_2025_745_MOESM1_ESM.docx]
